# Learning effects of virtual versus high-fidelity simulations in nursing students: a crossover comparison

**DOI:** 10.1186/s12912-022-00878-2

**Published:** 2022-04-27

**Authors:** SoMi Park, Hea Kung Hur, ChaeWeon Chung

**Affiliations:** 1 Department of Nursing, Wonju College of Medicine, Yonsei University, 20 Ilsan-ro, Wonju, Gangwon-do Korea; 2 College of Nursing, Research Institute of Nursing Science, Seoul National University, 103 Daehak-ro, Jongno-gu, Seoul, Korea

**Keywords:** Computer simulation, High-fidelity simulation, Learning, Maternity, Nursing

## Abstract

**Background:**

Simulation is an alternative or complementary method for students who cannot obtain sufficient direct care experience, as they allow students to experience various clinical situations. Mixed learning is becoming increasingly common as a way to provide students with opportunities to experience real-life clinical scenarios. This study compared the learning effects of a virtual simulation and a high-fidelity simulation in a different order of presentation, with a focus on training for premature rupture of membranes in the field maternity nursing. Through this comparison, this study aimed to obtain evidence to support decision-making regarding the most effective way to utilize mixed simulation strategies.

**Methods:**

A quasi-experimental, crossover-design study was performed with two randomly allocated groups of 26 junior nursing students each. The virtual simulation used the vSim® for nursing, and the high-fidelity simulation used a scenario developed by the research team. The learning effects were measured in terms of the problem-solving process, clinical reasoning, reflective thinking, satisfaction with the practicum, and self-confidence. The data collected with a structured questionnaire were analyzed using the Mann–Whitney test.

**Results:**

The virtual simulation-first, high-fidelity simulation-second order led to significantly higher scores for reflective thinking (z = 3.53, *p* < .001) and self-confidence (z = 2.47, *p* = .013) than the other order.

**Conclusions:**

The initial application of virtual simulation seemed to improve students’ thought processes, and then high-fidelity simulation seemed to allow them to perform actual practice better. Further trials of mixed learning methods are necessary to maximize learning effects in nursing education.

**Trial registration:**

KCT0005767 at 2021–01-12 registered.

## Background

Over the last 10 years, there has been a substantial increase in the use of simulations in undergraduate nursing education [1]. Simulation is an alternative or a complementary method for students who cannot obtain enough direct care experience, as they allow students to experience various clinical situations [2]. The National Council of State Boards of Nursing has acknowledged the effectiveness of simulation by stating that a portion of clinical hours could be replaced by simulation that target learning strategies [3]. Moreover, mixed learning has been widely utilized in recent years across higher education to produce better learning outcomes [4].

Previous studies of the mixed simulation were conducted with various topics, such as problem-based learning with high-fidelity simulation (HFS) for chronically ill patients [5] and emergency situations [6], combination of virtual simulations (VS) and HFS for children with asthma [7], and a simulation-based mastery program through iterative learning according to students’ competency in transfusion care [8]. However, most previous studies tended to compare a single simulation method to a mix of two methods. Thus, little is known regarding methods of combining multiple simulation methods or the most effective order of presentation when using a mixed simulation.

HFS is a technique that creates a situation or environment to allow persons to experience a representation of a real health care event with interactive computer mannikin for the purpose of practice, learning, evaluation, testing, or to gain understanding of systems or human actions [9]. The National Council of State Boards of Nursing study [3] found that HFS did not lead to different outcomes from those of a traditional method of clinical practicum, however, HFS continues to face limitations due to its time-consuming and labor-intensive nature, which has prompted recommendations to replace HFS with VS [10]. Nevertheless, a meta-analysis with 20 studies on applications of HFS to nursing education indicated that simulation education could improve learning outcomes in terms of knowledge, skill, and behavioral outcomes with a medium-to-large effect size, compared with traditional education [11].

VS provides a realistic world on the computer screen, offering dynamic and consistent experiences in a safe reproducible, accessible, and standardized clinical environment [12]. While a single HFS training session does not include repetitive practice, reassessment, and remediation, VS is thought to be sufficient to ensure a level of knowledge and skill required for clinical practice [13]. With the increasing utilization of VS, it became necessary to determine how best to use VS as a teaching strategy for learners. In a comparison of written case studies involving the use of VS by RN-BSN students, the VS intervention group reported higher scores for both satisfaction and confidence in learning [14]. In a recent meta-analysis with 12 studies [15], VR in nursing education was found to be more effective than the control conditions (traditional education methods and high or low fidelity simulations in improving knowledge. However, there was no difference between VR and the control conditions in skills, satisfaction, confidence, and performance time. Therefore, it is necessary to attempt mixed simulation to determine which method should be applied first to achieve more effective educational outcomes when applying simulation in nursing education.

Simulation-based learning for maternity nursing is a particularly high priority because clinical practice in this field is limited to observations of the care of women during pregnancy, parturition, and the neonatal period due to strict regulations regarding maternal rights and safety [16]. Thus, this study was initiated to examine the differences in learning effects according to the order of receiving VS and HFS in nursing students, with a particular focus on training for premature rupture of membrane (PROM) care, which is an important condition in maternity nursing.

## Methods

### Aims and design

**Fig. 1:**
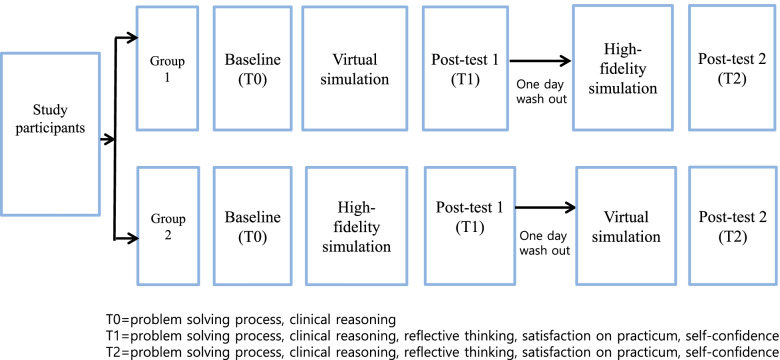
Research design

The study utilized a quasi-experimental, crossover design that applied VS and HFS in a different order to determine differences in 1) the problem-solving process, 2) clinical reasoning, 3) reflective thinking, 4) satisfaction with the practicum, and 5) self-confidence (Fig. 1).

The reason why the crossover design was chosen was, firstly, to identify which order would be more effective while allowing students to experience both methods; and secondly, to exclude any bias that could occur due to individual competency within the allocated group. In addition, the study used a consistent washout period of 1 day to minimize carry-over effects between the two groups.

### Participants

The inclusion criteria for participants were 1) students enrolled in the course of clinical practicum of maternity nursing in their junior year, 2) who had taken a lecture course on maternity nursing including PROM care, and 3) who voluntarily agreed to participate in the study and submitted a written consent form.

**Fig. 2:**
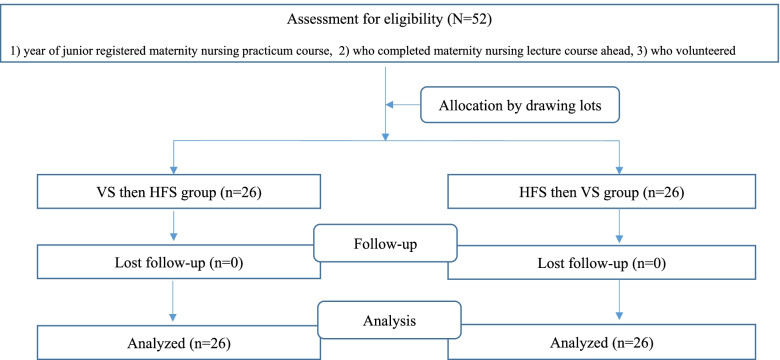
Study flow. VS, virtual simulation; HFS, high-fidelity simulation

Recruitment was done by a research assistant after explaining the purpose of the study and its process with a leaflet, and written consent was then obtained at the site. The sample size was calculated using G*Power 3.1.2 for the independent t-test with α=.05, 1-β=.80, and an effect size d=0.80 [17], for which 52 subjects were required (Fig. 2). Recruitment was closed when the sample size was reached by including the 52 students who had enrolled in the course. The median ages of the two groups were similar (22 vs. 21 years; z=0.89, *p*=.375), without a statistically significant difference.

Random allocation sequence was generated by the research team, and the random allocation was done by the research assistant using drawing lots. If a participant picked a red card, he/she was included in the group that received VS first and then HFS, while participants who picked a blue card started with HFS first and then received VS. Five small groups were composed for each group, and each small group consisted of 4–6 students. The participants involved in the study a total of 5 days for one time pre-test and two times of post-test with one-day wash out.

### Simulation for PROM nursing care

#### Virtual simulation

The VS for PROM nursing care utilized a web-based vSim® for nursing in a multimedia learning room with available personal computers. The program involved assessing a virtual patient, choosing an intervention, and then receiving prompt feedback immediately after the intervention. The scenario was developed through a collaboration between Wolter Kluwer Health, Laerdal Medical, and the National League for Nursing [18]. The VS employs a web-based platform to simulate the PROM scenario (a core case) whereby students have the opportunity to interact with a virtual patient. Students executed the simulation based on a protocol that consisted of safety measures, communication, assessment, intervention, drug and intravenous management, and testing and diagnosis. They then received feedback on their actions from the Laerdal program after completing the simulation. The VS in this study was also designed to be experiential and learner-centered; furthermore, learners could repeat the same scenario. Face-to-face debriefing, which was considered to be an important aspect of the program that would help students reflect upon their actions during the simulation [19], was then conducted for learners to share their experiences. The debriefing was composed of three phases which suggested by Mariani et al. [20]. First, in the descriptive phase, students shared their experiences while carrying out the simulation. Second, the analysis phase aimed to identify the current nursing problem presented in the simulation, prior nursing interventions, and missed assessments or interventions. Then, the final application phase included a discussion of how to apply the experiences to clinical practice.

#### High-fidelity simulation

It was developed to guide the implementation and evaluation of simulation education based on the Jeffries Simulation Theory [21]. It provides a nursing education strategy with major elements including the context, background, design, simulation experience, facilitator and educational strategies, participants, and outcomes [21]. The HFS was developed by the research team based on a standard of simulation module development [22]. The scenario was composed to guide students on how to use the personal information of a pregnant patient, to perform a health assessment, to communicate using the SBAR (Situation, Background, Assessment, Recommendation) tool, which was proposed by the Institute for Healthcare Improvement [23], to prepare for medical tests by checking doctor’s orders, and to perform a nursing intervention. The content validity of the scenario and algorithm of PROM nursing care [24] for the relevance and realism of the scenario was confirmed by a panel of a master's-level nurse with 10 years of clinical practice in a delivery center, an obstetrician, and three nursing faculty members who had taught clinical simulations for 10 years or more.

The HFS intervention with the topic of PROM nursing care was prepared to be done in an obstetrics ward room equipped with a patient simulator. The design of the actual simulation involved direct participation, observation of other students’ performances and one’s own recorded performance, and debriefing. Debriefing of the HFS was composed of the same phases with a face-to-face method.

### Simulation process

**Fig. 3:**
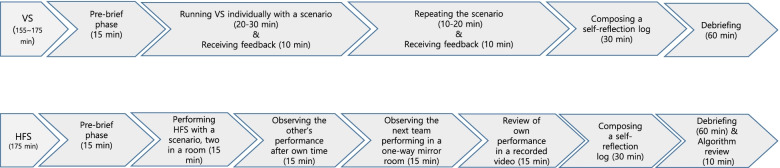
Process of Virtual simulation (VS) and High-fidelity simulation (HFS)

The actual process of the simulations is presented in Fig. 3. The intervention lasted for a total of 5 weeks. Two groups were invited to the intervention each week (one for VS then HFS and the other for HFS then VS, according to their allocation).

#### Virtual simulation

The coordinator of VS was a nurse with 5 years of clinical experience who had completed the vSim® for nursing education provided by the Laerdal Medical. She received information about the objectives of the practicum course and debriefing from the principal investigator of the study. The coordinator provided each group with an orientation as a pre-brief phase to make the students familiar with the operation methods and menu configuration and implementation of VS. She also helped students with any technical problems that occurred during the simulation. After repeated sessions of the scenario, students were assigned to write a reflection log according to the feedback provided by the Laerdal program. Next, a face-to-face debriefing with the principal investigator was conducted with 4 to 6 students at a time to share individuals’ experiences based on the reflection logs. Conducting the scenario took a final total of 155–175 min.

#### High-fidelity simulation

The three staff members for HFS consisted of a facilitator, who was a nurse in the Ph.D. program with more than 5 years of clinical and teaching experience, an operator, and a coordinator, who were also nurses with 3 years and 5 years of clinical experience, respectively.

The facilitator was responsible for giving cues to proceed with the scenario and assessing students’ responses according to the flow of the scenario. The operator of the software in the control room was in charge of presenting outcomes such as vital signs, uterine contractions, and lab data according to the flow of the scenario. She had received education about simulators and software of HFS from the manufacturer in advance. In addition, the coordinator played a role in giving an orientation on HFS and helping students’ observational learning. The principal investigator of the study led the HFS training for the staff, focusing on learning objectives, simulation scenarios, each individual’s tasks, and the debriefing process.

Two students entered the simulation room at the same time. One performed the HFS scenario as a nurse, then the other observed his/her acting. After switched roles with the same scenario, they moved to the one-way mirror room equipped with an audio–video system to observe the next team’s performance, followed by a review of one’s performance through the recorded video. Students then composed reflection logs, in which they compared their direct care with observed cases of others’ performance. Lastly, group debriefing by a face-to-face method with 4 to 6 members was performed and a summary of the PROM nursing care was delivered by the principal investigator through its algorithm. It took 175 min to operate the entire simulation. To prevent exposure of the scenarios within groups and between groups, students were alternatively allocated to VS and HFS before the invitation calls. Furthermore, the rooms for students were separated before and after the simulation to minimize mutual contact and information sharing.

### Data collection

Data collection was done from October 2020 through November 2020 by a research assistant. A baseline pre-test was done for all participants at the end of the week before starting the intervention in one of the seminar rooms, at which time students did not know which group they would be assigned to. Two post-tests were conducted, both in the debriefing room: after the first simulation (either VS or HFS) and after both simulations were completed. In the pre-test, two variables (the problem-solving process and clinical reasoning) were measured, and three additional variables (reflective thinking, satisfaction with the practicum, and self-confidence) were measured in the post-tests.

### Measurements

The instruments used for this research were as follows:

1) The problem-solving process referred to learners’ thinking process, which was measured by the Problem-Solving Process Behavior Survey developed by Park and Woo [25]. This 5-point scale consists of 25 items, and a high value indicates higher problem-solving ability. As an indicator of its reliability, Park and Woo [25] reported a Cronbach’s α of 0.89, and Cronbach’s α was 0.88 in this study.

2) Clinical reasoning referred to the capability of decision-making in response to a nursing problem by assessing the patient’s data and was measured using the Korean version of the Nurses Clinical Reasoning Scale (NCRS) scale translated by Joung and Han [26], on which a high score on a 5-point scale of 15 questions indicates high clinical reasoning capability. Cronbach’s α was 0.93 in Joung and Han's [26] study, and it was 0.91 in this study.

3) Reflective thinking, meaning self-evaluation and recognition of one's performance, was measured using the Reflective Learning Continuum developed by Peltier et al. [27]. Out of 25 questions used by Koo [28] for nurses, 16 items that reflected learners’ perceptions of acquiring new learning were chosen for measurements on a 4-point scale. For this instrument, higher scores correspond to a higher level of reflective thinking. The reliability was shown by a Cronbach’s α of 0.86 in Koo [28]’s study, and Cronbach’s α was 0.85 in this study.

4) Satisfaction with the practicum referred to the satisfaction level of the students who participated in the simulation practice experience, and it was measured by 5 items on a 5-point scale provided by National League for Nursing [29], which had been translated into Korean in the study of Hur et al. [30]. A higher score of the measured values on this scale corresponds to a higher level of satisfaction with the practicum. Cronbach’s α in the study of Hur et al. [30] was 0.87, while it was 0.92 in this study.

5) Self-confidence refers to students’ confidence for nursing implementation, and it was measured by 8 items on a 5-point scale provided by National League for Nursing [29], which had also been used in the study of Hur et al. [30]. A higher score indicates higher confidence after the simulation experience. Cronbach’s α was 0.75 in the study of Hur et al. [30] and it was 0.73 in this study.

### Data analysis

Data were analyzed using IBM SPSS Statistics 25.0, and an α level of 0.05 was set using the two-tailed test.

Since the number of samples for each group in this study was 26, normality and variance tests were performed for each group. According to the Shapiro–Wilk test for normality, D(26) ranged from 0.77 to 0.95 (*p* = 0.001–0.448) for the group that received VRS first and HFS second, and D(26) ranged from 0.79 to 0.96 (*p* = 0.001–0.363) for the group that received HFS first and VRS second, indicating that some data were not normally distributed.

Thus, the differences in the learning effects according to the treatment order were analyzed using the Mann–Whitney U test. The effect size was calculated since a nonparametric test was performed.

### Ethical considerations

Institutional review board approval (No. CR320055) was obtained before the implementation of this study and the study was registered in the Clinical Research Information Service of Korea Disease Control and Prevention Agency (http://cris.nih.go.kr/cris/index/index.do) (No. KCT0005767, 12/01/2021). The Consolidated Standards of Reporting Trials (CONSORT) 2010 Statement (http://www.consort-statement.org/consort-2010) was followed while composing this report, and the completed CONSORT checklist is available in Supplemental File 1. Informed consent was obtained from all the study participants. They were informed about voluntary participation and the right to withdraw from the study at any time. To ensure confidentiality, any identifying information of the participants was removed and numbered identifiers were used. In particular, it was emphasized to enrollees in the practicum course that there were no disadvantages of not participating in the study, and the same simulation programs were provided to students who did not participate. It was also explained that participants’ answers would only be used for research purposes and would not be included in the course grade. In addition, to avoid any possible influence on students, the principal investigator participated in neither the simulation process nor data collection.

## Results

### Differences in learning effects by a treatment order

At the initial time point, there were no statistically significant between-group differences in clinical reasoning (z = 0.35, *p* = 0.728) or the problem-solving process (z = 1.01, *p* = 0.314).

The group that received VS first and HFS second showed significantly higher scores for reflective thinking (z = 3.52, *p* < 0.001), with a Cohen’s D for an effect size of 0.91, and higher self-confidence (z = 2.47, *p* = 0.013), with a Cohen’s D of 0.72.

After the first simulation session, a significant difference was also seen in clinical reasoning (z = 2.16, *p* = 0.031) and problem-solving process (z = 2.76, *p* = 0.006), with higher scores in the group that performed VS than in those that performed HFS, with Cohen’s D values of 0.82 and 0.72, respectively (Table 1).

**Table 1:** Differences of the Learning Effects by Time Point and by Treatment Order of VS and HFS (*N* = 52)

Time/Group	Baseline (T0)			After 1^st^ treatment (T1)			After 2^nd^ Treatment (T2)		Crossover effect (T2-T1)		
Variables (possible range)	Group1 (*n* = 26)	Group2 (*n* = 26)	z (*p*)	Group1 (*n* = 26)	Group2 (*n* = 26)	z (*p*)	Group1 (*n* = 26)	Group2 (*n* = 26)	Group1 (*n* = 26)	Group2 (*n* = 26)	z (*p*)
	Median (IQR)			Median (IQR)			Median (IQR)		Median (IQR)		
Problem solving process (25 ~ 125)	84.5 (17.50)	78.5 (10.00)	1.01 (.314)	92.5 (12.75)	84.5 (10.00)	2.76 (.006)	98.0 (11.25)	93.0 (10.00)	5.5 (11.00)	9.5 (7.25)	-1.30 (.193)
Clinical reasoning (15 ~ 75)	48.0 (9.50)	48.5 (10.00)	0.35 (.728)	56.0 (7.00)	52.5 (8.25)	2.16 (.031)	60.5 (5.50)	57.0 (4.50)	5.0 (7.25)	5.0 (6.50)	0.00 (1.000)
Reflective thinking (16 ~ 64)	NA	NA	NA	52.0 (7.75)	56.0 (9.25)	-1.05 (.295)	60.0 (6.25)	57.0 (10.00)	6.0 (5.25)	1.5 (5.00)	3.52 (< .001)
Satisfaction on practicum (16 ~ 64)	NA	NA	NA	22.5 (4.25)	24.0 (5.00)	-2.00 (.842)	25.0 (2.00)	23.0 (5.00)	1.0 (3.00)	0.0 (1.50)	1.81 (.070)
Self-confidence (8 ~ 40)	NA	NA	NA	33.0 (5.25)	33.0 (4.00)	0.35 (.726)	37.5 (4.00)	34.0 (4.25)	3.0 (6.25)	0.5 (2.75)	2.47 (.013)

*VS* Virtual simulation, *HFS* High-fidelity simulation

Group1: VS then HFS, Group2: HFS then VS

*IQR* Interquartile range

## Discussion

Nursing educators must constantly consider and strive to implement effective teaching and learning methods so that students obtain the required nursing competence to meet the various needs faced in the nursing field. Above all, nursing educators should ensure that students are ready to practice nursing in today’s complex clinical environment [31]. Therefore, this study used a crossover design to improve the learning effects of simulations by applying a different treatment order of VS and HFS to a group of students with the same clinical case.

Simulation education emphasizes providing realistic conditions by utilizing computerized patient simulators, virtual reality, and standardized patients [32] to help learners achieve a better level of practical capabilities. The results of this study revealed that the learners who performed VS first and HFS second showed higher reflective thinking and self-confidence than those who engaged in simulations in the other order. Reflective thinking during VS is thought to be facilitated by prompt feedback and repeated learning [33]; moreover, the personalized feedback log in vSim® for nursing is considered to help learners increase their reflective thinking [34]. It provides not only essential part of intervention needed in the section of main opportunities for improvement, but also the student’s performance records in the basic and detailed view section to present what she/he performed or not in addition with patient’s data. Thus, its application as the initial simulation would have influenced the students’ learning outcomes. In addition, the outcome of increased self-confidence was consistent with previous results [35] because learners would feel less stress when carrying out VS according to the individual learner’s level of performance. Consequently, VS worked in advance to assist learners’ thinking flow, which helped them assess the clinical situation and select an intervention to prioritize, and it served as pre-learning for HFS practice in which knowledge, skills, and attitudes were integrated, thereby enhancing self-confidence.

Furthermore, when examining the effects of each simulation (HFS vs. VS) after the first treatment in each group, the students in the VS first group showed significantly higher scores for clinical reasoning and the problem-solving process than those in the HFS group. As consistent with previous findings, VS was useful in improving the prioritization of nursing care [36] and students’ clinical judgment [37]. VS has been viewed favorably in healthcare education and has the potential to help learners with independent knowledge development, critical thinking and problem solving [38, 39]. As Jenson and Forsyth [40] pointed out, this is because VS uses a computer-based program to simulate a virtual patient and healthcare environment that responds to assessment and interventions performed by the students by selecting the appropriate actions on the computer.

A meaningful aspect of this study is that VS and HFS were used as two complementary methods to deliver education on the same topic (PROM nursing care). It is also thought that the repeated activities in VS and HFS, such as learning by video, peer observation, and using self-evaluation seemed to work together to motivate the students. This simulation education focused on learner-oriented paradigms [21], which helped them gain confidence in practical performance through repeated learning.

A couple of limitations should be noted. First, the use of a sample from one nursing school might limit the sample representativeness and ability to make inferences. Second, this study was implemented as part of a maternity nursing practicum course and was conducted in line with the overall practicum schedule. Thus, the diffusion of the intervention could not be completely controlled.

## Conclusion

It is time to think about not only how to overcome the limitations of the clinical environment of observation-oriented clinical practice, but also how to conduct simulation training in nursing education in the more complex clinical situation in the post-COVID-19 era. Future consumers of education, referred to as generation MZ, are thought to be more familiar with modern information and communication technology, which requires us to be prepared for their educational needs.

Thus, this study, which was the first to attempt a crossover design to investigate this issue, confirmed that mixed learning in an order of VS first and HFS second could provide transformative learning experiences. It is recommended to utilize VS first, as it provides opportunities to enhance students’ learning effects in a non-contact, safe environment using computers or mobile devices. The application of simulations should be expanded by conducting various mixed simulation methods.

## Data Availability

The datasets used and analyzed during the current study are available from the corresponding author on reasonable request.

## Abbreviations

VS: Virtual simulation
HFS: High-fidelity simulation
PROM: Premature rupture of membrane

## References

[1] Doolen J, Mariani B, Atz T, Horsley TL, Rourke JO, McAfee K (2016). High-fidelity simulation in undergraduate nursing education: a review of simulation reviews. Clin Simul Nurs.

[2] Kim JH, Park IH, Shin S (2013). Systematic review of Korean studies on simulation within nursing education. J Korean Acad Soc Nurs Educ.

[3] Hayden JK, Smiley RA, Alexander M, Kardong-Edgren S, Jeffries PR (2014). The NCSBN national simulation study: a longitudinal, randomized, controlled study replacing clinical hours with simulation in prelicensure nursing education. J Nurs Regul.

[4] Dziuban C, Graham CR, Moskal PD, Norberg A, Sicilia N (2018). Blended learning: the new normal and emerging technologies. Int J Educ Technol High Educ.

[5] Yun SY, Choi JY (2019). A comparative study on learning outcomes according to the integration sequences of S-PBL in nursing students: Randomized crossover design. J Korean Acad Nurs.

[6] Liaw SY, Chen FG, Klainin P, Brammer J, O'Brien A, Samarasekera DD (2010). Developing clinical competency in crisis event management: an integrated simulation problem-based learning activity. Adv Health Sci Educ Theory Pract.

[7] Kim M, Kim S, Lee WS (2019). Effects of a virtual reality simulation and a blended simulation of care for pediatric patient with asthma. Child Health Nurs Res.

[8] Park SM, Song HY, Hur HK, Kim KK (2020). Effects of simulation-based mastery learning of blood transfusion for nursing students. J Korean Soc Simul Nurs..

[9] Lioce L, Lopreiato J, Downing D, Chang TP, Robertson JM, Anderson M (2020). Healthcare Simulation Dictionary, Publication No. 20–0019.

[10] Shaikh F, Inayat F, Awan O, Santos MD, Choudhry AM, Waheed A (2017). Computer-assisted learning applications in health educational informatics: A review. Cureus..

[11] Shin S, Park JH, Kim JH (2015). Effectiveness of patient simulation in nursing education: meta-analysis. Nurse Educ Today.

[12] Cobbett S, Snelgrove-Clarke E (2016). Virtual versus face-to-face clinical simulation in relation to student knowledge, anxiety, and self-confidence in maternal-newborn nursing: a randomized controlled trial. Nurse Educ Today.

[13] Solvik E, Struksnes S (2018). Training nursing skills: a quantitative study of nursing students’ experiences before and after clinical practice. Nurs Res Pract.

[14] Turrise SL, Thompson CE, Hepler M (2020). Virtual simulation: Comparing critical thinking and satisfaction in RN-BSN students. Clin Simul Nurs.

[15] Chen FQ, Leng YF, Ge JF, Wang DW, Li C, Chen B (2020). Effectiveness of virtual reality in nursing education: meta-analysis. J Med Internet Res.

[16] Lee SH (2019). Trend analysis of research in the journal of Korean society for simulation in nursing over a 6-year period (2013–2018). J Korean Soc Simul Nurs.

[17] Padilha JM, Machado PP, Ribeiro A, Ramos J, Costa P (2019). Clinical virtual simulation in nursing education: randomized controlled trial. J Med Internet Res.

[18] Laerdal Medical and Wolters Kluwer Health. vSim for Nursing Maternity. https://thepoint.lww.com/gateway (2021). Accessed 1 May, 2021.

[19] Kim YJ, Yoo JH (2020). The utilization of debriefing for simulation in healthcare: a literature review. Nurse Educ Pract.

[20] Mariani B, Cantrell MA, Meakim C, Prieto P, Dreifuerst KT (2013). Structured debriefing and students' clinical judgment abilities in simulation. Clin Simul Nurs.

[21] Jeffries PR, Rodgers B, Adamson K (2015). NLN Jeffries simulation theory: Brief narrative description. Nurs Educ Perspect.

[22] Korea Accreditation Board of Nursing Education. Standard for simulation practice. http://old.kabone.or.kr/HyAdmin/upload/goodFile/120170421113900.pdf (2017). Accessed 11 April, 2021.

[23] Institute for Healthcare Improvement. SBAR: Situation-Background-Assessment-Recommendation. http://www.ihi.org/Topics/SBARCommunicationTechnique/Pages/default.aspx (2021). Accessed 19 Aug 2021.

[24] Rundell K, Panchal B (2017). Preterm labor: prevention and management. Am Fam Physician.

[25] Park JW, Woo OH (1999). The effects of PBL (Problem-BasedLearning) on problem solving process by learner's metacognitive level. J Educ Technol.

[26] Joung J, Han JW (2017). Validity and reliability of a Korean version of nurse clinical reasoning competence scale. J Korea Acad Ind Coop Soc.

[27] Peltier JW, Hay A, Drago W (2005). The reflective learning continuum: Reflecting on reflection. J Mark Educ.

[28] Koo JS (2016). Mediating effect of reflection on the relationship between work motivation and nursing competency according to the nursing clinical ladder among staff nurses working in general hospital. Master's thesis.

[29] National League for Nursing. Student satisfaction and self-confidence in learning. https://www.nln.org/docs/default-source/uploadedfiles/default-document-library/instrument-2-satisfaction-and-self-confidence-in-learning.pdf (2005). Accessed 15 Aug, 2020.

[30] Hur HK, Shin YH, Park S, Lim YM, Kim GY, Kim KK (2014). Effectiveness of an emergent care management simulation education among senior nursing students according to learning styles. J Korea Contents Assoc.

[31] Ulrich D, Farra S, Smith S, Hodgson E (2014). The student experience using virtual reality simulation to teach decontamination. Clin Simul Nurs.

[32] Ross JG, Latz E, Meakim CH, Arcamone A, Reynolds K (2022). Multiple-patient simulations and student outcomes in prelicensure nursing education: an integrative review. Clin Simul Nurs.

[33] Verkuyl M, Lapum JL, St-Amant O, Hughes M, Romaniuk D, McCulloch T (2020). Exploring debriefing combinations after a virtual simulation. Clin Simul Nurs.

[34] Kim MJ, Kang HS, De Gagne JC (2021). Nursing students’ perceptions and experiences of using virtual simulation during the COVID-19 pandemic. Clin Simul Nurs.

[35] Kim JI, Kang H, Park S, Ahn S (2014). Current status of women's health nursing practicum and direction. Korean J Women Health Nurs.

[36] Foronda CL, Fernandez-Burgos M, Nadeau C, Kelley CN, Henry MN (2020). Virtual simulation in nursing education: a systematic review spanning 1996 to 2018. Simul Healthc.

[37] Fogg N, Kubin L, Wilson CE, Trinka M (2020). Using virtual simulation to develop clinical judgment in undergraduate nursing students. Clin Simul Nurs.

[38] Allaire JL (2015). Assessing critical thinking outcomes of dental hygiene students utilizing virtual patient simulation: a mixed methods study. J Dent Educ.

[39] Sunnqvist C, Karlsson K, Lindell L, Fors U (2016). Virtual patient simulation in psychiatric care – a pilot study of digital support for collaborate learning. Nurse Educ Pract.

[40] Jenson CE, Forsyth DM (2012). Virtual reality simulation: using three-dimensional technology to teach nursing students. Comput Inform Nurs.

